# Prophylactic octreotide does not reduce the incidence of postoperative chylothorax following lobectomy: Results from a retrospective study

**DOI:** 10.1097/MD.0000000000016599

**Published:** 2019-07-19

**Authors:** Chu Zhang, Hui Zhang, Wenbin Wu, Dong Liu, Dunpeng Yang, Miao Zhang, Cuntao Lu

**Affiliations:** a Department of Thoracic Surgery, Shaoxing People's Hospital (Shaoxing Hospital, Zhejiang University School of Medicine), Shaoxing; b Department of Thoracic Surgery, Xuzhou Central Hospital Affiliated to Southeast University, Xuzhou, China.

**Keywords:** chylothorax, lung cancer, octreotide, pleural effusion, readmission

## Abstract

Chylothorax after lobectomy is common, lacking reliable preventive measures. Octreotide is widely used for treatment of chyle leakage, but its role in preventing chylothorax has not been estimated. The aim of this study was to evaluate whether prophylactic octreotide could reduce the incidence of postoperative chylothorax.

Patients who underwent lobectomy for lung cancer from January 2016 to September 2018 were retrospectively reviewed. The cases in prophylactic group received octreotide 1 day before the surgery until removal of chest tubes, while those in the control group did not use it unless the diagnosis of chylothorax.

A total of 379 patients were enrolled, with 190 patients in control and 189 cases in prophylactic group. Octreotide was well tolerated in patients who received this agent. No 30-day mortality was indicated. Seven cases in control (3.7%, 7/190) and 3 cases in prophylactic group (1.6%, 3/189) with chylothorax were observed (*P* = .337). The patients in prophylactic group showed shorter duration of chest drainage ([3.6 ± 1.6] days vs [4.1 ± 2.0] days, *P* = .006) and reduced drainage volume ([441.8 ± 271.1] mL vs [638.7 ± 463.3] mL, *P* < .001). In addition, they showed similar stations and numbers of dissected lymph nodes, surgery-related complications, and postoperative hospital stay. Besides, 11 (5.8%, 11/190) patients in control and 6 (3.2%, 6/189) cases in the prophylactic group were readmitted for pleural effusion needing reinsertion of chest tubes (*P* = .321). Moreover, multivariable logistic analysis showed that induction therapy (odds ratio [OR] =12.03; 95% confidence interval [CI] 3.15–46.03, *P* < .001) was a risk factor, while high-volume experience of the surgeon (OR = 0.23; 95% CI 0.06–0.97, *P* = .045) was a preventive factor of surgery-related chylothorax. Additionally, prophylactic octreotide (OR = 0.18; 95% CI 0.11–0.28, *P* < .001) and perioperative low-fat diet (OR = 0.46; 95% CI 0.29–0.73, *P* = .001) were negatively associated with the drainage volume of pleural effusion. Furthermore, high-volume experience of the surgeon (OR = 6.03; 95% CI 1.30–27.85, *P* = .021) and induction therapy (OR = 8.87; 95% CI 2.97–26.48, *P* < .001) were risk factors of unplanned readmission.

Prophylactic octreotide does not reduce the incidence of postoperative chylothorax or unplanned readmission following anatomic lobectomy. The routine application of octreotide should not be recommended. High-quality trials are required to validate these findings.

## 1 Introduction

Chylothorax after pulmonary resection and systemic lymph node dissection (SLND) occurs in 1.4% of the patients.^[1]^ The elucidation of predictive factors of surgery-related chylothorax is helpful to establish reasonable perioperative management. Octreotide is effective in the treatment of chylous leakage.^[2]^ To the best of our knowledge, whether prophylactic octreotide could reduce the incidence of postoperative chylothorax has not been reported before.

Herein, a retrospective cohort study in 2 hospitals was conducted to evaluate the preventive role of octreotide against chylothorax following lobectomy for lung cancer. Besides, correlated factors of postoperative chylothorax, pleural effusion, and unplanned readmission of the patients were also investigated.

## 2 Patients and methods

### 2.1 Inclusion and exclusion criteria for data collection

This retrospective cohort study was approved by the Institutional Review Board and the Ethics Committee of Xuzhou Central Hospital affiliated to Southeast University and Shaoxing People's Hospital (Shaoxing Hospital, Zhejiang University School of Medicine). Informed consent was obtained from every patient who was enrolled in this study. The cases undergoing video-assisted thoracoscopic (VATS) lobectomy and mediastinal SLND for non-small cell lung cancer (NSCLC) between January 2016 and September 2018 in our institutions were reviewed. The inclusion criteria were:

(1) the tumor was localized without distal metastasis on contrast-enhanced computed tomography (CT) or positron emission tomography images, and
(2) their American Society of Anesthesiologists score, left ventricular ejection fraction, and pulmonary function was appropriate for anatomic major pulmonary resection.

The exclusion criteria were:

(1) previous chest surgery or other cancer history,
(2) thoracic duct ligation or resection during the surgery for any reasons, and
(3) the informed consent was unavailable.

Patients who were allergic to octreotide or had a history of allergy to anything were enrolled into the control group.

### 2.2 Prophylactic octreotide and low-fat diet

The patients in control group received normal perioperative treatment without octreotide, until the diagnosis of chylothorax when it was used for treatment. The cases in prophylactic group received octreotide injection (0.1 mg every 8 hours) 1 day before the surgery, until the removal of chest tubes. However, the surgeons chose patients to receive prophylactic octreotide mainly according to their personal experience in terms of the management of lobectomy-related chylous leakage, lacking specific selection criteria. The major considerations of octreotide usage were:

(1) the tumor invaded mediastinal tissue, and
(2) the CT images showed fusion of enlarged mediastinal lymph nodes, as the surgery was expected to be difficult.

Besides, the octreotide administration was avoided in those patients with a history of allergy to somatostatin or its analogs. Meanwhile, 73 (38.4%) cases in control group and 78 (41.3%) cases in prophylactic group started low-fat oral diet (fat intake <10 g/d) 1 week before the operation until removal of chest tubes. Similarly, these patients were chosen to take special diet just in accordance with the experience of the clinicians, without standard selection criteria, while the others took normal diet unless the occurrence of chylothorax when low-fat diet was utilized as therapy.

### 2.3 Enhanced recovery after surgery protocol

Fast-track protocol in thoracic surgery was performed individually. Medical or physical pulmonary rehabilitation was conducted 1 to 2 weeks before surgery. Smoking cessation was mandatory for smokers. Oral carbohydrate-containing clear fluid was administered 6 and 2 hours before the operation, respectively.

Postoperative multimodal analgesia was applied to improve pain relief and promote early mobilization,^[3]^ including ultrasound-guided serratus anterior plane block using bupivacaine. Neostigmine and opioids were avoided. The urethral catheter was removed after their recovery from general anesthesia. Besides, mobilization out of bed and oral feeding were initiated 6 hours after the surgery. Then the chest tube in patients without chylothorax or bleeding was removed at a threshold of 200 to 300 mL per 24 hours,^[4]^ after the exclusion of atelectasis or significant pleural effusion by chest X-ray. As for patients with chylothorax, their chest tubes were removed if the effluent remains ≤50 mL per day.

### 2.4 Preoperative simulation of anatomical resection

Three dimensional-computed tomography bronchography and angiography (3D-CTBA) was performed preoperatively using the free software of OsiriX,^[5]^ with the aim to diminish the accidental bleeding during the surgery. Anatomic lobectomy simulation was conducted accordingly, followed by a detailed resection plan to effectively handle the variations of pulmonary vessels.

### 2.5 Surgical procedures

The surgery was performed under general anesthesia, with single-lung ventilation through a double-lumen endotracheal tube. The surgical access was protected by a plastic soft-tissue retractor with the aim to reduce trauma on the intercostal nerve and diminish contamination of the surgical site. Milk or Oliver oil to facilitate the identification of thoracic duct was not applied in this cohort. Meanwhile, prophylactic en masse ligation of the thoracic duct, mechanical or chemical pleurodesis was not performed. The surgical incision was made without rib spreading, except for pneumonectomy. For uniportal VATS, a 4-cm incision was made in the 4th intercostal space for resection of upper lobe or the 5th intercostal space for middle or lower lobe, between the anterior and middle axillary line. For 2-port VATS, another 1-cm observation incision was made through the 7th intercostal space along the middle axillary line. Pulmonary vessels, bronchi, and incomplete fissure were divided sequentially with endoscopic staplers, followed by mediastinal SLND. One 24 or 26 French tube was inserted in the posterior part of the incision for postoperative chest drainage.

In detail, conventional uniportal VATS lobectomy was performed along the sequence of pulmonary artery, vein, and bronchus using endoscopic linear staplers.^[6]^ If the artery is invisible in fissures, the procedure could be performed from bottom to top, with fissure stapling as the final step. Single-direction VATS was another procedure for major lung resection. The resection of upper and middle lobes proceeded in a single direction from the ventrum to the dorsum, while the resection of the lower lobes proceeded in a caudal to cranial direction, with the aim to avoid repeated overturn and retraction of the lung.^[7]^ For example, single-direction uniportal VATS left upper lobectomy was carried out following the sequence of the anterior segmental artery, superior pulmonary vein, the recurrent branch from left main pulmonary artery, left upper bronchus, the posterior and lingular segmental artery, and the fissure.^[8]^ Besides, right upper lobectomy was performed from the front to the back of the patient.^[9]^ Dissections of the lymph nodes were finally manipulated.

The operations were performed by different surgeons in 2 hospitals, whose experience was graded as low-volume (<100 lobectomies per year) and high-volume (≥200 lobectomies per year).

### 2.6 Identification and management of chylothorax

Chylothorax was suspected when there was excessive chest drainage (>400 mL/d) with or without an odorless milky appearance. The chylous leakage was confirmed by presence of triglycerides (>110 mg/dL) and/or appearance of chylomicrons with positive Sudan III staining in the pleural effusion. The chylothorax cases were initially treated conservatively using octreotide (0.3 mg/d) in combination with low-fat diet. If the daily chylous fluid exceeded 500 mL for 5 days after conservative treatment, surgical intervention would be considered.

### 2.7 Follow up

The patients were followed up for at least 3 months after the surgery. CT was performed when the patient showed dyspnea after discharge. Re-insertion of a chest tube would be considered if the CT image indicated newly-emerged mild to moderate volume of pleural effusion. Primary endpoints of this study were the incidence of postoperative chylothorax and unplanned readmission, while the secondary endpoints were chest drainage duration and overall drainage volume after lobectomy.

### 2.8 Statistical analysis

Continuous data of clinical parameters were presented as means ± standard deviations. Statistical analysis was performed using Statistical Package for the Social Sciences software version 23.0 for Windows (SPSS Inc., Chicago, IL). Student *t* test or Wilcoxon test was used to compare continuous data, while Chi-square or Fisher exact test was utilized for dichotomous or categorical variables. Risk factors for postoperative chylothorax, readmission, and pleural effusion were assessed by stepwise multivariate logistic regression analysis, with chylothorax, readmission, and pleural effusion as the dependent variable respectively, and significance set at the 0.05 level. A 2-sided *P*-value of <.05 was considered statistically significant.

## 3 Results

### 3.1 Patient demographics

**Figure 1:**
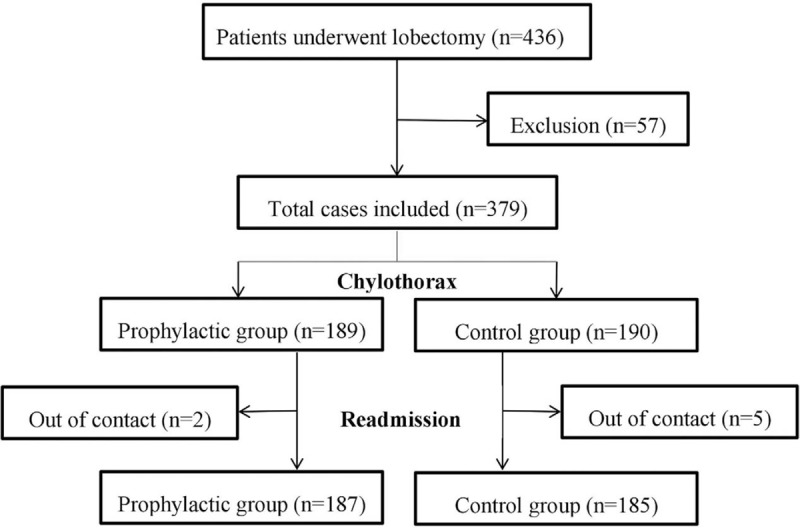
The ischemic illustration of data collection in this study.

From January 2016 to September 2018, there were 436 patients who underwent major lung resection and SLND for primary NSCLC at our institutions. Finally, the data of 379 cases who met the inclusion criteria were reviewed, with 231 male and 148 female cases, at a mean age of 62.3 years (range, 32–85 years). The ischemic illustration of data collection was indicated in Figure 1. Comorbidities including hypertension, diabetes mellitus, coronary heart disease, chronic obstructive pulmonary diseases, emphysema, and bronchiectasis, as well as smoking history were recorded. There was no significant difference in age (*P* = .243), gender (*P* = .401), body mass index (BMI, *P* = .845), T stage (*P* = .178), comorbidities (*P* = .088), smoking history (*P* = .758), low-fat diet (*P* = .601), and distribution of experienced surgeons (*P* = .469) between the 2 groups, except neoadjuvant therapy (*P* = .046) and tumor location (*P* = .003), as shown in Table 1.

**Table 1:**
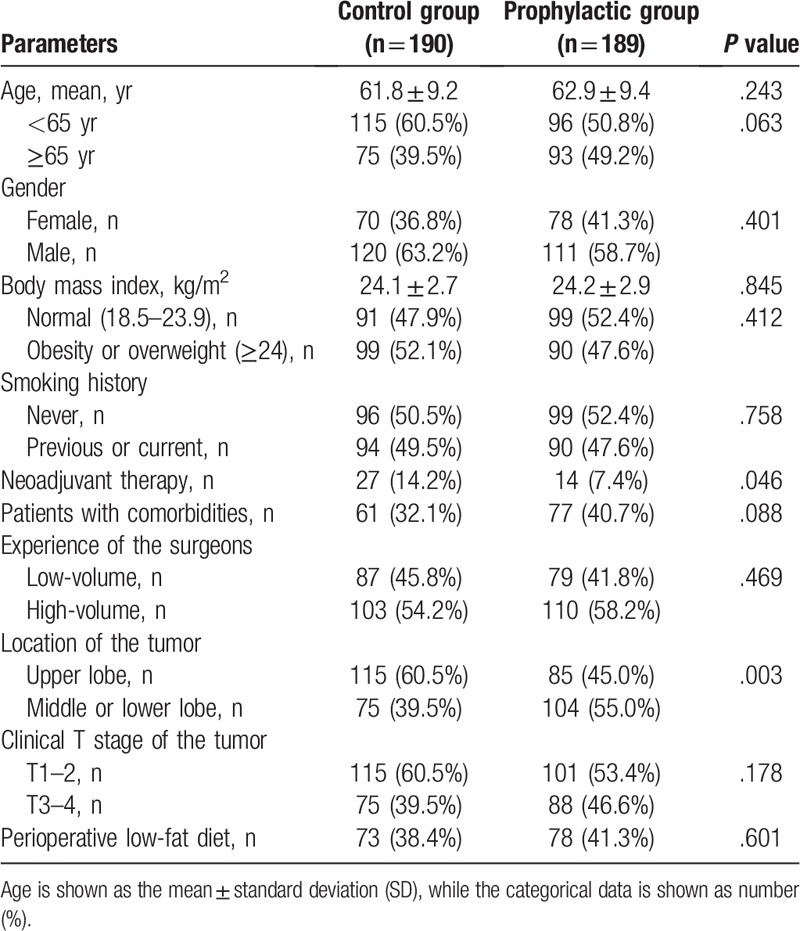
Baseline profile of the patients.

### 3.2 Operative features

All the operations were performed successfully without injury of pulmonary or bronchial vessels. Thirty-day mortality, major blood loss, bronchopleural fistula, heart failure, respiratory failure, pulmonary embolism, or deep vein thrombosis was not indicated in this cohort. As shown in Table 2, 74 (38.9%) cases in control and 88 (46.6%) cases in prophylactic group performed resection simulation using 3D-CTBA digital anatomic models (*P* = .147). There were 38 (20.0%), 65 (34.2%), and 87 (45.8%) patients underwent surgery using 2-portal, uniportal, and single-direction uniportal VATS procedures in the control group, respectively. Accordingly, there were 20 (10.6%), 71 (37.6%), and 98 (51.8%) cases underwent 2-portal, uniportal, and single-direction uniportal VATS in the prophylactic group, with a significant difference between the groups (*P* = .039).

**Table 2:**
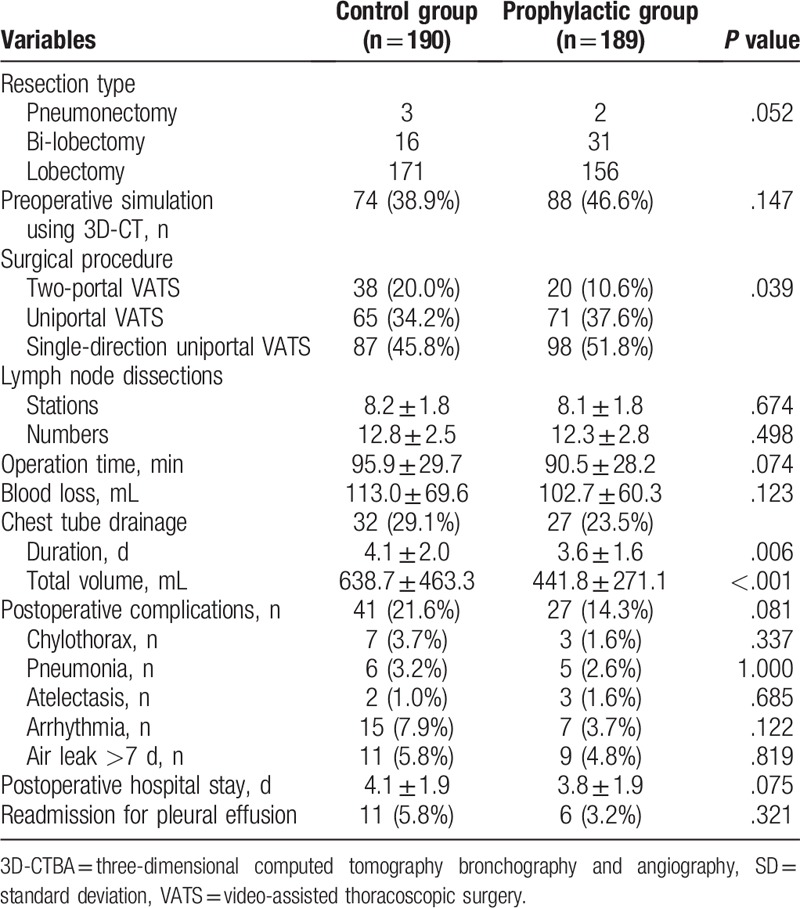
Operative details.

However, as compared with the control, the patients in the prophylactic group showed significantly shorter chest tube duration ([3.6 ± 1.6] days vs [4.1 ± 2.0] days, *P* = .006), and less overall drainage volume ([441.8 ± 271.1] mL vs [638.7 ± 463.3] mL, *P* < .001) after surgery. However, significant difference was not indicated in terms of operation time ([95.9 ± 29.7] min vs [90.5 ± 28.2] min, *P* = .074), blood loss ([113.0 ± 69.6] mL vs [102.7 ± 60.3] mL, *P* = .123), the stations of dissected lymph nodes ([8.2 ± 1.8] vs [8.1 ± 1.8], *P* = .074), complications (41 [21.6%] vs 27 [14.3%], *P* = .081), as well as postoperative hospital stay ([4.1 ± 1.9] days vs [3.8 ± 1.9] days, *P* = .074). No significant side effect that necessitated discontinuation of octreotide was observed.

### 3.3 Incidence and management of chylothorax

A total of 10 chylothorax cases (2.6%, 10/379) were reported in this cohort, with a median age of 60.6 years (range, 42–77). The incidence of chylothorax was similar in prophylactic and control group (1.6% [3/189] vs 3.7% [7/190], *P* = .337). Besides, 5 of these 10 cases received preoperative chemotherapy, while 2 of them received radiotherapy before surgery. These patients were cured after conservative treatment by low-fat diet and octreotide, without surgical intervention.

### 3.4 Unplanned readmission after discharge

During the follow-up, 11 patients (5.8%, 11/190) in the control and 6 (3.2%, 6/189) in the prophylactic group were readmitted, because of residual pleural effusion on CT images, without a statistical difference (*P* = .321). Among them, 3 cases showed moderate pleural effusion (500–1000 mL) while the other 14 cases showed mild pleural effusion (<500 mL), as shown in Table 3. After a re-insertion of a 7 French chest catheter, they were cured within 1 to 3 days. Delayed chylothorax was not indicated in these cases.

**Table 3:**
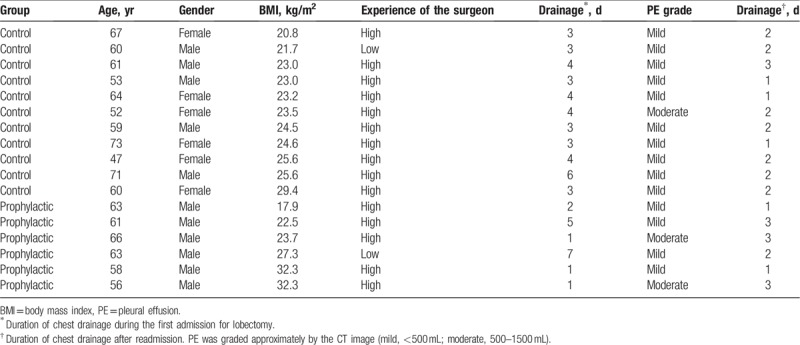
The readmitted 17 patients for residual pleural effusion needing reinsertion of chest tubes.

### 3.5 Logistic regression analysis on risk factors of chylothorax, pleural effusion, and unplanned readmission

First, variables of perioperative characteristics were selected in multivariate logistic analysis, with the aim to identify potential risk factors of postoperative chylothorax. Statistical significance was not indicated in terms of age (≥65 years vs <65 years, *P* = .874), gender (female vs male, *P* = .304), BMI (obesity or overweight vs normal or lean, *P* = .555), comorbidity (yes vs no, *P* = .379), smoking history (yes vs no, *P* = .958), T stage of the tumor (T3–4 vs T1–2, *P* = .825), tumor location (middle or lower lobe vs upper lobe, *P* = .361), perioperative low-fat diet (yes vs no, *P* = .490), prophylactic octreotide (yes vs no, *P* = .435), surgical procedures (uniportal VATS vs 2-portal VATS, *P* = .192), 3D-CTBA simulation (*P* = .476), operation time (*P* = .690), stations (*P* = .533) or numbers (*P* = .281) of dissected lymph nodes. As shown in Table 4, preoperative chemotherapy or radiotherapy (odds ratio [OR] = 12.03; 95% confidence interval [CI] 3.15–46.03, *P* < .001) was estimated to be a risk factor of chylothorax, while high-volume experience of the surgeon (OR = 0.23; 95% CI 0.06–0.97, *P* = .045) was a preventive factor of surgery-related chylothorax.

**Table 4:**
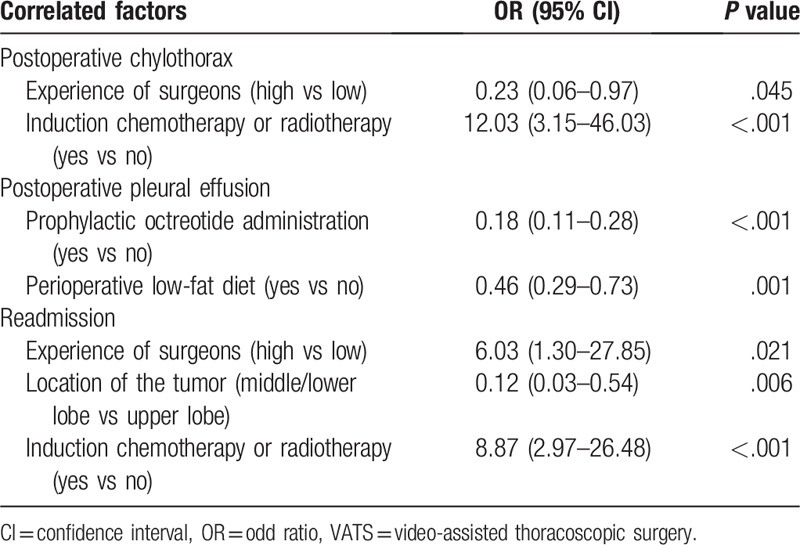
Multivariate logistic analysis on the correlated risk factors.

Next, another multivariate logistic analysis was conducted to estimate risk factors of postoperative pleural effusion. Significant correlation was not shown regarding age (*P* = .066), gender (*P* = .103), BMI (*P* = .718), smoking history (*P* = .754), location (*P* = .583), or T stage (*P* = .079) of the tumor, neoadjuvant therapy (*P* = .185), comorbidity (*P* = .859), surgical procedures (*P* = .169), 3D-CTBA simulation (*P* = .412), operation time (*P* = .919), stations (*P* = .614), or numbers of dissected lymph nodes (*P* = .279). Furthermore, prophylactic octreotide (OR = 0.18; 95% CI 0.11–0.28, *P* < .001), and perioperative low-fat diet (OR = 0.46; 95% CI 0.29–0.73, *P* = .001) were preventive factors of the pleural effusion volume, as shown in Table 4.

Furthermore, 17 cases in this cohort study were readmitted within 1 month after their first discharge. Among them, eight cases received induction chemotherapy or radiotherapy. Statistical significance correlated with readmission was not indicated in age (*P* = .541), gender (*P* = .705), BMI (*P* = .538), smoking history (*P* = .449), T stage (*P* = .337), comorbidity (*P* = .562), perioperative low-fat diet (*P* = .921), prophylactic octreotide (*P* = .732), surgical procedures (*P* = .185), 3D-CTBA simulation (*P* = .144), chylothorax (*P* = .299), blood loss (*P* = .765), or postoperative hospital stay (*P* = .320). As shown in Table 4, high-volume experience of the surgeon (OR = 6.03; 95% CI 1.30–27.85, *P* = .021), and neoadjuvant therapy (OR = 8.87; 95% CI 2.97–26.48, *P* < .001) were risk factors of unplanned readmission for pleural effusion. On the contrary, tumor location (OR = 0.12; 95% CI 0.03–0.54, *P* = .006) was negatively correlated with readmission.

## 4 Discussion

The prevention of postoperative chylothorax following lobectomy is a clinical challenge. As an effective therapeutic agent for chyle leakage, octreotide might have a role in reducing the incidence of this potentially lethal complication. However, efficacy data is lacking. As shown in this study, prophylactic octreotide is not associated with decreased incidence of chylothorax after anatomic lung resection and SLND or unplanned readmission for residual pleural effusion, although it could decrease the total volume of postoperative pleural effusion. In addition, neoadjuvant therapy is both a risk factor of chylothorax and readmission following lobectomy. Based on these findings, although octreotide with low-fat diet is the first-line therapeutic choice for chylous leakage following thoracic surgery, it does not display a preventive efficacy. Accordingly, there are several issues need to be elucidated.

Chylothorax leads to compromised immune function through continuous loss of nutrition and lymphocytes of the patients,^[10]^ so preventive methods for reducing the incidence of postoperative chylous leakage are essential. It is reported that oral milk before surgery decreases the risk of iatrogenic injury to the thoracic duct.^[11]^ Nevertheless, preventive thoracic duct ligation does not seem to reduce the incidence of chylothorax.^[12]^ Failure of surgical intervention might be ascribed to extensive damage of thoracic duct following surgery or radiotherapy.

Perioperative octreotide, a long-acting somatostatin analog having an inhibitory action on pancreatic exocrine secretion, is proposed to reduce the incidence of postoperative pancreatic fistula, which has been widely used in the last 2 decades.^[13]^ However, an updated meta-analysis shows that prophylactic somatostatin analogs do not improve the postoperative outcomes or reduce the incidence of pancreatic fistula following pancreaticoduodenectomy.^[14]^ Patients presenting with a history of gut neuroendocrine tumors or carcinoid syndrome can experience life-threatening carcinoid crises during anesthesia or surgery, and continuous infusions of high-dose octreotide can minimize intraoperative carcinoid crises.^[15]^ However, another study indicates that octreotide infusions do not prevent intraoperative carcinoid crises in carcinoid patients.^[16]^

Octreotide could decrease the intestinal absorption of fats and triglyceride concentration in the thoracic duct,^[17]^ and it is effective at slowing down chylous leakage. Surgical intervention is not always effective,^[10]^ whereas octreotide shows an acceptable safety profile.^[18,19]^ A review indicates that octreotide is useful in 47% of patients with chylothorax.^[20]^ It is mainly administrated subcutaneously at a dosage of 0.05 to 0.2 mg, 3 times a day. A high-dose of octreotide (intravenous 0.25 mg, 3 times/d for 14 days) in a patient with very large chylous after failed band ligation shows satisfactory efficacy, which indicates that early adoption of octreotide might be superior to surgical intervention.^[17]^ The general consensus for conservative management of chylothorax with octreotide is 1 week before surgical intervention.^[21]^ Besides, the adverse events of octreotide include but not limited to gastrointestinal disturbances such as nausea, vomiting, loose stool, flatulence, and abdominal pain, in addition, liver dysfunction, biliary stasis, gallstones, hypoglycemia, and hyperglycemia have also been reported in children patients,^[22]^ while another study shows that the adverse effects of octreotide are reported in 14.3% of patients.^[20]^ In our study, nineteen patients (10.1%) showed nausea during octreotide administration.

Analysis on large national databases shows that the readmission rate within 30 days after pulmonary lobectomy is 12%.^[23]^ Meanwhile, the unplanned readmission rate after lobectomy for stage I lung cancer is 6%, without difference between thoracotomy and VATS.^[24]^ Readmission after lobectomy with or without fast-tracking is 5.5%.^[25]^ Overall, readmission rates seem to vary between 5% and 12%. The readmission rate within 30 days after lobectomy and SLND in our study is 4.5% (17/379), which is consistent with the previous reports. Reasons for unplanned readmission or return to outpatient clinic include wound infection, pneumonitis or pneumothorax, pleural infection or effusion, fever, chest pain, and generalized muscle weakness, while nearly 50% of them are ascribed to pulmonary or pleural complications.^[26]^ There is a concern that a shorter hospital stay may be correlated with increased risk of readmission.^[27]^ Although a shorter length of hospital stay might result in premature discharge, our study showed that neither length of postoperative hospital stay nor chylothorax is predictor of unplanned readmission. Furthermore, it is not correlated with smoking history, comorbidities, 3D-CTBA simulation, or surgical approaches in our study. An evaluation based on large database shows that the incidence of readmission is similar in thoracotomy, VATS lobectomy, and pneumonectomy, and the most common cause of readmission is pulmonary-related reasons, mostly 1 week after discharge.^[28]^ Unplanned transfer to the intensive care unit and higher comorbidity score predicts the risk of readmission.^[26]^ Therefore, readmission could not be ascribed to a shorter hospital stay, which is one of the major parameters of fast-track thoracic surgery.

It is unclear whether there is a preventive method (eg, smoking cessation or diabetic control) can reduce the risk of readmission in lobectomy patients.^[29,30]^ Consequently, the identification of preventive factors of chylothorax and readmission needs further research. As shown in this cohort, high-volume experience of the surgeon is a protective factor of postoperative chylothorax, which might indicate that chylothorax is mainly a technical issue. Besides, most of these patients have an uneventful recovery period. However, the detailed aspects of VATS lobectomy and SLND could not be taught easily to young surgeons. Besides, the readmission does not necessarily reflect the quality of care.^[26]^ It is noteworthy that, the high-volume experience of surgeons is an independent risk factor of readmission in our study. Experienced clinicians might prefer shorter operation time and faster discharge, which might result in unreasonable removal of chest tubes. Nevertheless, better clinical evidence is necessary to confirm this deduction.

Fissureless technique in lobectomy can significantly decrease the development of prolonged air leak and time to air leak cessation after pulmonary lobectomy.^[31]^ Besides, as sequentially resection of superficial pulmonary veins, deep bronchi and deeper pulmonary arteries, and fissure in a single direction does not require repeated turnover of the lobes,^[7]^ single-direction procedure for thoracoscopic lobectomy is utilized by many experienced surgeons, with the aim to shorten operation time.

### 4.1 Limitations of this study

First, this cohort study is limited by its small sample size and retrospective nature, and few cases of chylothorax as well as unplanned readmissions. Second, this review is conducted from 2 institutions by thoracic surgeons with different experiences and skills. Furthermore, standard criteria for patient selection to receive octreotide or low-fat diet were lacking. There is also a selection bias regarding the preferred choice of surgical approach and fast-track protocol. In the era of precision medicine, because very little is known about the role of prophylactic intervention on chylothorax and unplanned readmission after thoracic surgery, high-quality researches are warranted.

## 5 Conclusion

In summary, prophylactic octreotide should not be applied routinely for patients undergoing lobectomy, as it does not decrease the incidence of postoperative chylothorax or unplanned readmission. Large-scale and randomized controlled studies with long-term follow up of the patients are necessary to verify our findings.

## Author contributions

**Conceptualization:** Dong Liu, Miao Zhang.

**Data curation:** Hui Zhang, Dong Liu, Dunpeng Yang, Miao Zhang.

**Formal analysis:** Chu Zhang, Cuntao Lu.

**Funding acquisition:** Dong Liu, Cuntao Lu.

**Investigation:** Cuntao Lu.

**Methodology:** Chu Zhang, Hui Zhang, Dunpeng Yang, Miao Zhang.

**Project administration:** Dong Liu, Cuntao Lu.

**Software:** Hui Zhang, Miao Zhang.

**Supervision:** Miao Zhang.

**Validation:** Cuntao Lu.

**Visualization:** Hui Zhang.

**Writing – original draft:** Chu Zhang, Wenbin Wu, Dunpeng Yang, Miao Zhang, Cuntao Lu.

**Writing – review and editing:** Chu Zhang, Wenbin Wu, Miao Zhang.
